# PSYCHIATRIC HISTORY AND LATER-LIFE COGNITIVE CHANGE: EFFECT MODIFICATION BY SEX, RACE, AND ETHNICITY

**DOI:** 10.1093/geroni/igac059.2091

**Published:** 2022-12-20

**Authors:** Maria Brown, Miriam Mutambudzi

**Affiliations:** Syracuse University, Syracuse, New York, United States; Syracuse University, Syracuse, New York, United States

## Abstract

**Objective:**

To better understand life course influences affecting cognitive function and decline in later life, we explored sex and race/ethnicity differentials in the relationship between a history of psychiatric, emotional, or nervous problems and cognitive functioning in later life, while accounting for early life disadvantage and relevant covariates.

**Methods:**

Multi-level growth curve models examined associations between psychiatric history and cognitive functioning, and differences by sex and race/ethnicity (SRE), in 20,155 Health and Retirement Study (1995-2014) participants aged 65 or older, by estimating cognition scores and plotting trajectories of change with age by SRE.

**Results:**

A history of psychiatric, emotional, or nervous problems was significantly related to cognition scores and rates of decline. Hispanic and Black participants had significantly lower cognition scores at age 75 and steeper rates of decline than White females, and Black race and the Hispanic race-sex interaction erased the protective effects of being female.

**Conclusions:**

Our findings indicate that members of minority groups with a history of psychiatric problems evidence lower cognitive function in later life, and as a result, have a greater need for community-based long-term care than their peers without this history. Future research should include longitudinal analyses of different components of cognitive function, specific psychiatric diagnoses, and life history data that capture socioeconomic and psychosocial experiences throughout the life course. Population level findings as reported here, along with aggregate findings from similar studies, can inform interventions and policies regarding support for populations that are vulnerable to mental illness and to subsequent cognitive decline.

